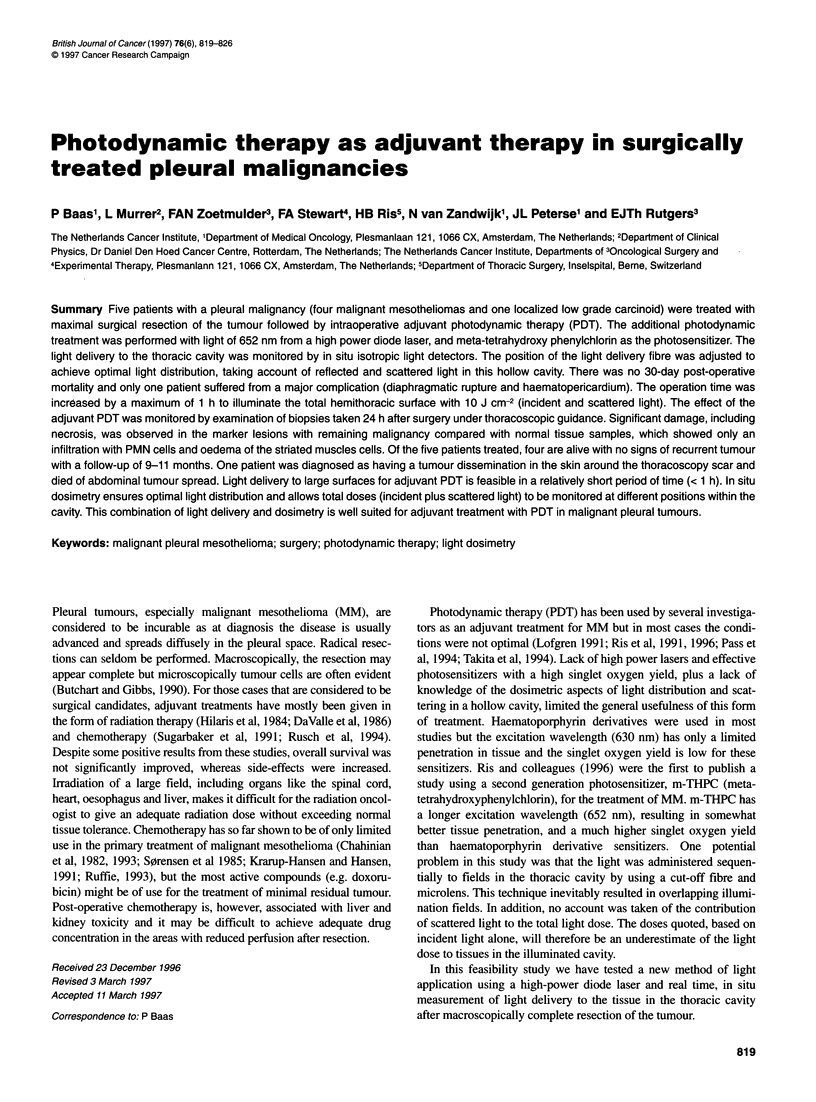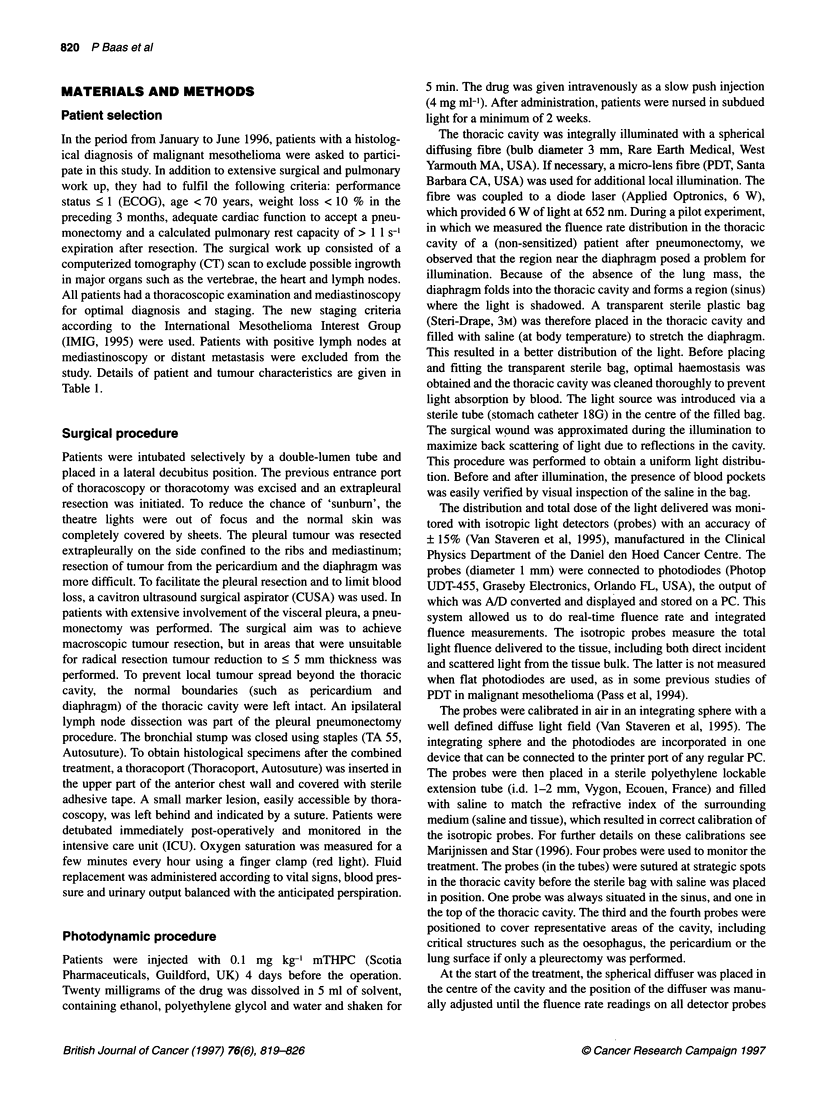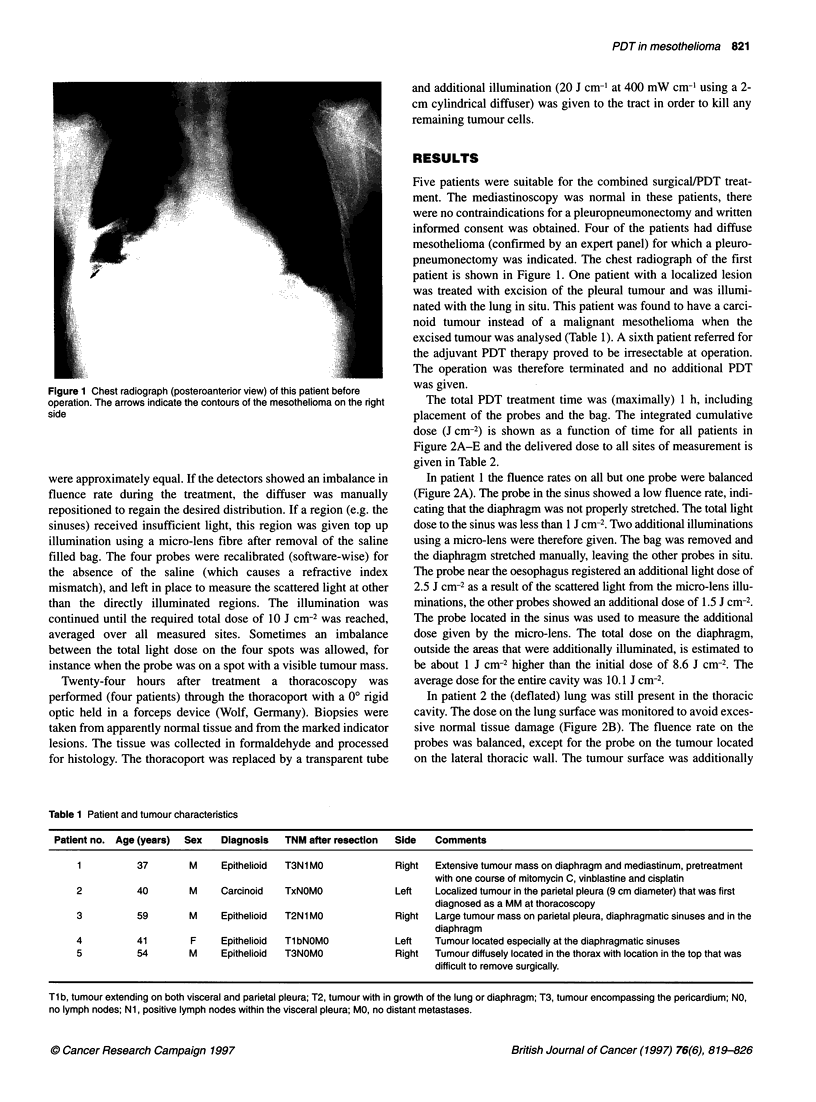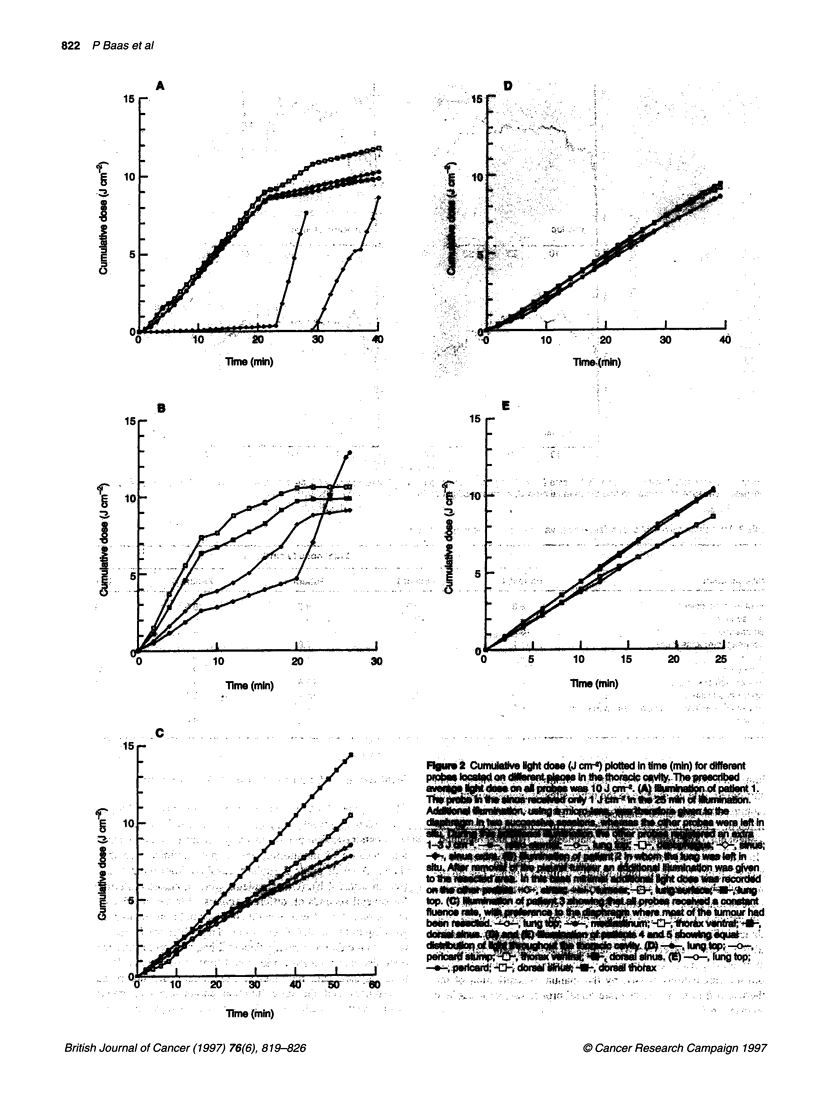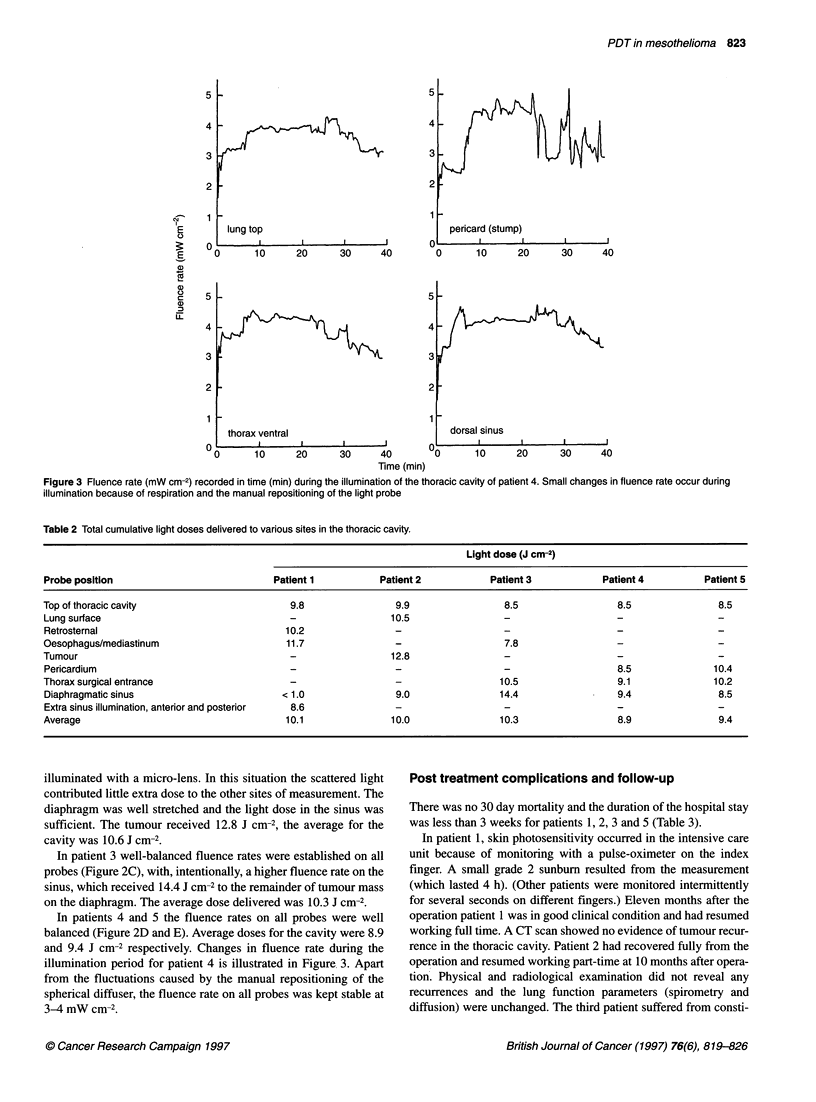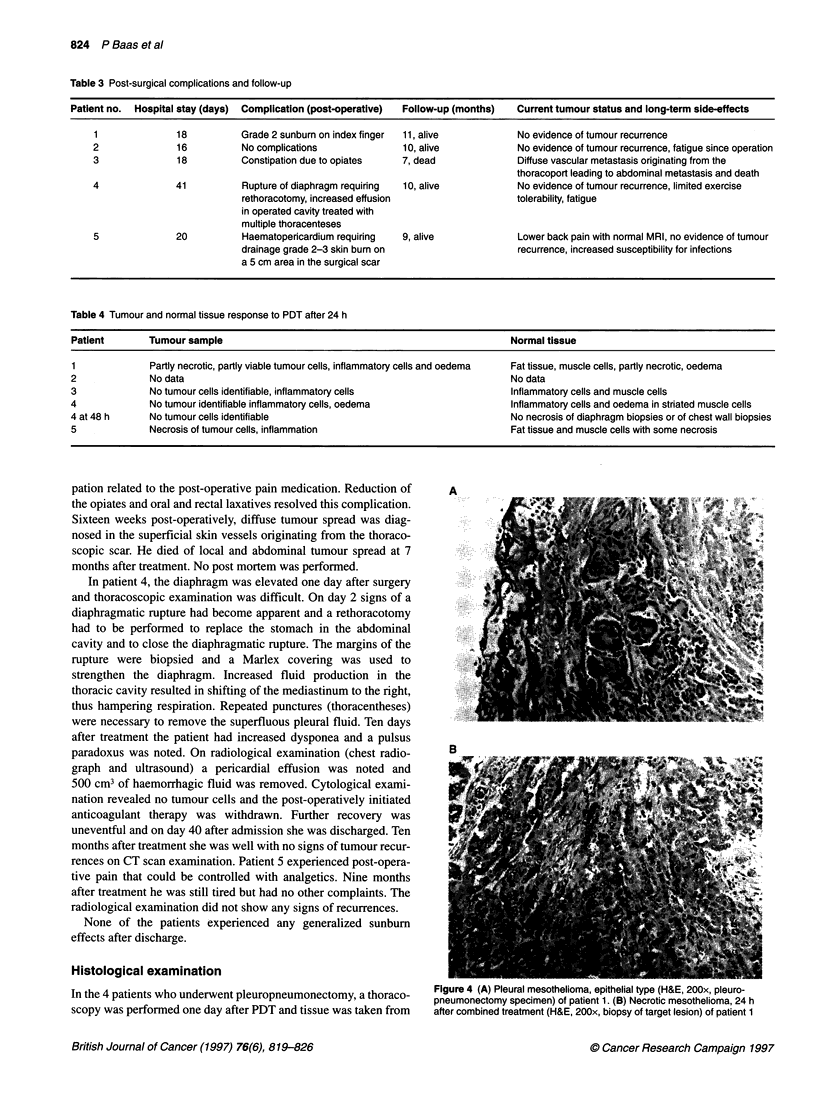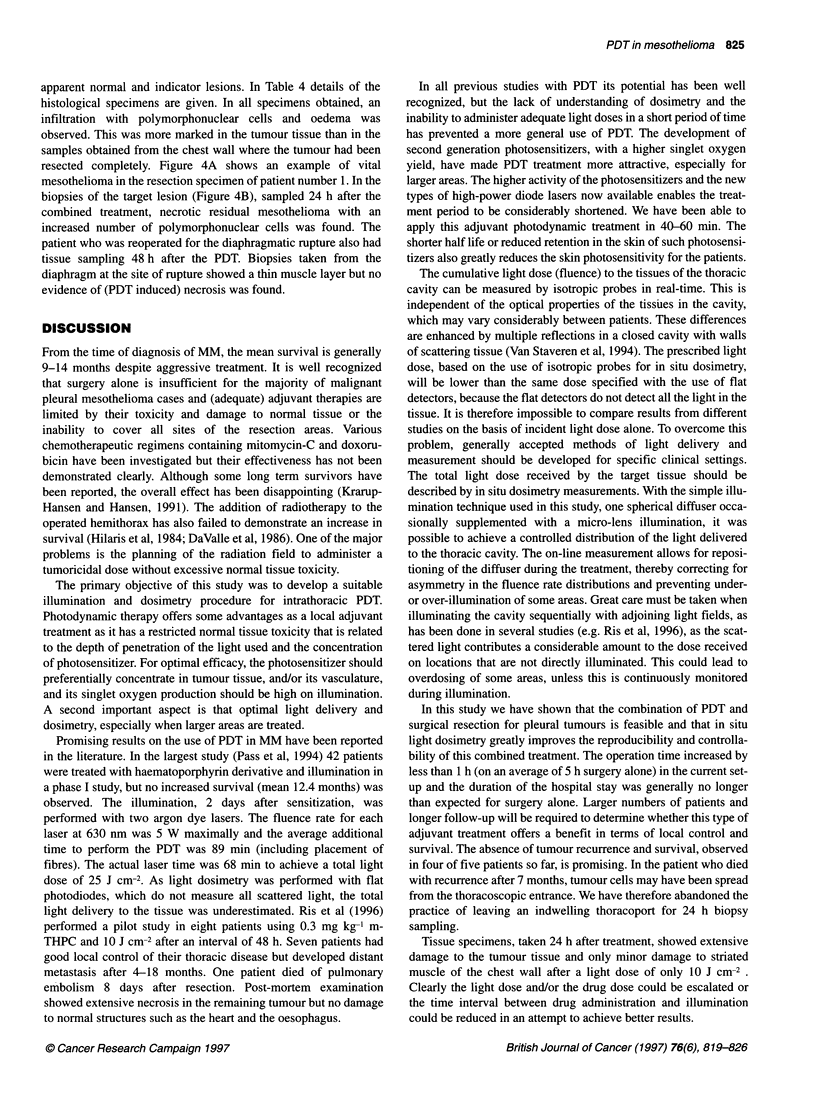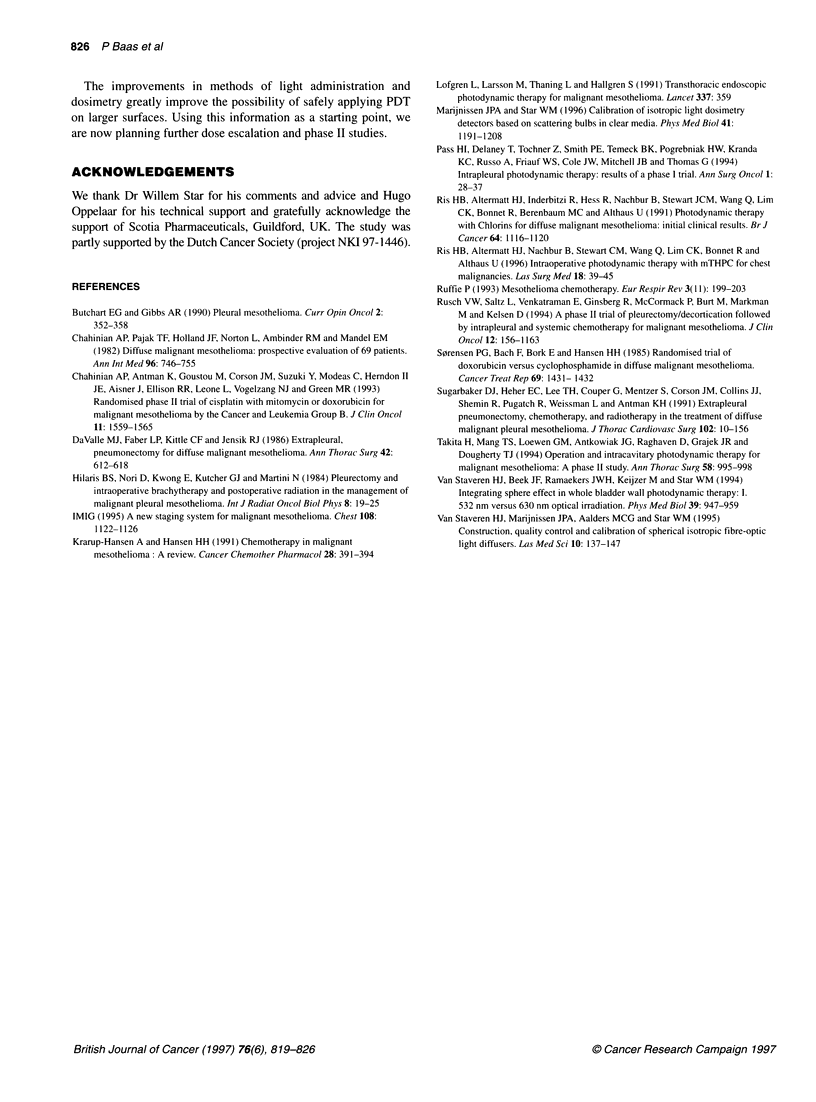# Photodynamic therapy as adjuvant therapy in surgically treated pleural malignancies.

**DOI:** 10.1038/bjc.1997.468

**Published:** 1997

**Authors:** P. Baas, L. Murrer, F. A. Zoetmulder, F. A. Stewart, H. B. Ris, N. van Zandwijk, J. L. Peterse, E. J. Rutgers

**Affiliations:** The Netherlands Cancer Institute, Department of Medical Oncology, Amsterdam.

## Abstract

**Images:**


British Journal of Cancer (1997) 76(6), 819–826
© 1997 Cancer Research Campaign

### Photodynamic therapy as adjuvant therapy in surgically treated pleural malignancies

**P Baas[1], L Murrer[2], FAN Zoetmulder[3], FA Stewart[4], HB Ris[5], N van Zandwijk[1], JL Peterse[1] and EJTh Rutgers[3]**

The Netherlands Cancer Institute, [1]Department of Medical Oncology, Plesmanlaan 121, 1066 CX, Amsterdam, The Netherlands; [2]Department of Clinical Physics, Dr Daniel Den Hoed Cancer Centre, Rotterdam, The Netherlands; The Netherlands Cancer Institute, Departments of [3]Oncological Surgery and [4]Experimental Therapy, Plesmanlann 121, 1066 CX, Amsterdam, The Netherlands; [5]Department of Thoracic Surgery, Inselspital, Berne, Switzerland

**Summary** Five patients with a pleural malignancy (four malignant mesotheliomas and one localized low grade carcinoid) were treated with maximal surgical resection of the tumour followed by intraoperative adjuvant photodynamic therapy (PDT). The additional photodynamic treatment was performed with light of 652 nm from a high power diode laser, and meta-tetrahydroxy phenylchlorin as the photosensitizer. The light delivery to the thoracic cavity was monitored by in situ isotropic light detectors. The position of the light delivery fibre was adjusted to achieve optimal light distribution, taking account of reflected and scattered light in this hollow cavity. There was no 30-day post-operative mortality and only one patient suffered from a major complication (diaphragmatic rupture and haematopericardium). The operation time was incréased by a maximum of 1 h to illuminate the total hemithoracic surface with 10 J $cm^{-2}$ (incident and scattered light). The effect of the adjuvant PDT was monitored by examination of biopsies taken 24 h after surgery under thoracoscopic guidance. Significant damage, including necrosis, was observed in the marker lesions with remaining malignancy compared with normal tissue samples, which showed only an infiltration with PMN cells and oedema of the striated muscles cells. Of the five patients treated, four are alive with no signs of recurrent tumour with a follow-up of 9–11 months. One patient was diagnosed as having a tumour dissemination in the skin around the thoracoscopy scar and died of abdominal tumour spread. Light delivery to large surfaces for adjuvant PDT is feasible in a relatively short period of time (< 1 h). In situ dosimetry ensures optimal light distribution and allows total doses (incident plus scattered light) to be monitored at different positions within the cavity. This combination of light delivery and dosimetry is well suited for adjuvant treatment with PDT in malignant pleural tumours.

**Keywords:** malignant pleural mesothelioma; surgery; photodynamic therapy; light dosimetry

Pleural tumours, especially malignant mesothelioma (MM), are considered to be incurable as at diagnosis the disease is usually advanced and spreads diffusely in the pleural space. Radical resections can seldom be performed. Macroscopically, the resection may appear complete but microscopically tumour cells are often evident (Butchart and Gibbs, 1990). For those cases that are considered to be surgical candidates, adjuvant treatments have mostly been given in the form of radiation therapy (Hilaris et al, 1984; DaValle et al, 1986) and chemotherapy (Sugarbaker et al, 1991; Rusch et al, 1994). Despite some positive results from these studies, overall survival was not significantly improved, whereas side-effects were increased. Irradiation of a large field, including organs like the spinal cord, heart, oesophagus and liver, makes it difficult for the radiation oncologist to give an adequate radiation dose without exceeding normal tissue tolerance. Chemotherapy has so far shown to be of only limited use in the primary treatment of malignant mesothelioma (Chahinian et al, 1982, 1993; Sørensen et al 1985; Krarup-Hansen and Hansen, 1991; Ruffie, 1993), but the most active compounds (e.g. doxorubicin) might be of use for the treatment of minimal residual tumour. Post-operative chemotherapy is, however, associated with liver and kidney toxicity and it may be difficult to achieve adequate drug concentration in the areas with reduced perfusion after resection.

*Received 23 December 1996*
*Revised 3 March 1997*
*Accepted 11 March 1997*

*Correspondence to:* P Baas

Photodynamic therapy (PDT) has been used by several investigators as an adjuvant treatment for MM but in most cases the conditions were not optimal (Lofgren 1991; Ris et al, 1991, 1996; Pass et al, 1994; Takita et al, 1994). Lack of high power lasers and effective photosensitizers with a high singlet oxygen yield, plus a lack of knowledge of the dosimetric aspects of light distribution and scattering in a hollow cavity, limited the general usefulness of this form of treatment. Haematoporphyrin derivatives were used in most studies but the excitation wavelength (630 nm) has only a limited penetration in tissue and the singlet oxygen yield is low for these sensitizers. Ris and colleagues (1996) were the first to publish a study using a second generation photosensitizer, m-THPC (meta-tetrahydroxyphenylchlorin), for the treatment of MM. m-THPC has a longer excitation wavelength (652 nm), resulting in somewhat better tissue penetration, and a much higher singlet oxygen yield than haematoporphyrin derivative sensitizers. One potential problem in this study was that the light was administered sequentially to fields in the thoracic cavity by using a cut-off fibre and microlens. This technique inevitably resulted in overlapping illumination fields. In addition, no account was taken of the contribution of scattered light to the total light dose. The doses quoted, based on incident light alone, will therefore be an underestimate of the light dose to tissues in the illuminated cavity.

In this feasibility study we have tested a new method of light application using a high-power diode laser and real time, in situ measurement of light delivery to the tissue in the thoracic cavity after macroscopically complete resection of the tumour.

### MATERIALS AND METHODS

### Patient selection

In the period from January to June 1996, patients with a histological diagnosis of malignant mesothelioma were asked to participate in this study. In addition to extensive surgical and pulmonary work up, they had to fulfil the following criteria: performance status ≤ 1 (ECOG), age < 70 years, weight loss < 10 % in the preceding 3 months, adequate cardiac function to accept a pneumonectomy and a calculated pulmonary rest capacity of > 1 l $s^{-1}$ expiration after resection. The surgical work up consisted of a computerized tomography (CT) scan to exclude possible ingrowth in major organs such as the vertebrae, the heart and lymph nodes. All patients had a thoracoscopic examination and mediastinoscopy for optimal diagnosis and staging. The new staging criteria according to the International Mesothelioma Interest Group (IMIG, 1995) were used. Patients with positive lymph nodes at mediastinoscopy or distant metastasis were excluded from the study. Details of patient and tumour characteristics are given in Table 1.

### Surgical procedure

Patients were intubated selectively by a double-lumen tube and placed in a lateral decubitus position. The previous entrance port of thoracoscopy or thoracotomy was excised and an extrapleural resection was initiated. To reduce the chance of 'sunburn', the theatre lights were out of focus and the normal skin was completely covered by sheets. The pleural tumour was resected extrapleurally on the side confined to the ribs and mediastinum; resection of tumour from the pericardium and the diaphragm was more difficult. To facilitate the pleural resection and to limit blood loss, a cavitron ultrasound surgical aspirator (CUSA) was used. In patients with extensive involvement of the visceral pleura, a pneumonectomy was performed. The surgical aim was to achieve macroscopic tumour resection, but in areas that were unsuitable for radical resection tumour reduction to ≤ 5 mm thickness was performed. To prevent local tumour spread beyond the thoracic cavity, the normal boundaries (such as pericardium and diaphragm) of the thoracic cavity were left intact. An ipsilateral lymph node dissection was part of the pleural pneumonectomy procedure. The bronchial stump was closed using staples (TA 55, Autosuture). To obtain histological specimens after the combined treatment, a thoracoport (Thoracoport, Autosuture) was inserted in the upper part of the anterior chest wall and covered with sterile adhesive tape. A small marker lesion, easily accessible by thoracoscopy, was left behind and indicated by a suture. Patients were detubated immediately post-operatively and monitored in the intensive care unit (ICU). Oxygen saturation was measured for a few minutes every hour using a finger clamp (red light). Fluid replacement was administered according to vital signs, blood pressure and urinary output balanced with the anticipated perspiration.

### Photodynamic procedure

Patients were injected with 0.1 mg $kg^{-1}$ mTHPC (Scotia Pharmaceuticals, Guildford, UK) 4 days before the operation. Twenty milligrams of the drug was dissolved in 5 ml of solvent, containing ethanol, polyethylene glycol and water and shaken for 5 min. The drug was given intravenously as a slow push injection (4 mg $ml^{-1}$). After administration, patients were nursed in subdued light for a minimum of 2 weeks.

The thoracic cavity was integrally illuminated with a spherical diffusing fibre (bulb diameter 3 mm, Rare Earth Medical, West Yarmouth MA, USA). If necessary, a micro-lens fibre (PDT, Santa Barbara CA, USA) was used for additional local illumination. The fibre was coupled to a diode laser (Applied Optronics, 6 W), which provided 6 W of light at 652 nm. During a pilot experiment, in which we measured the fluence rate distribution in the thoracic cavity of a (non-sensitized) patient after pneumonectomy, we observed that the region near the diaphragm posed a problem for illumination. Because of the absence of the lung mass, the diaphragm folds into the thoracic cavity and forms a region (sinus) where the light is shadowed. A transparent sterile plastic bag (Steri-Drape, 3M) was therefore placed in the thoracic cavity and filled with saline (at body temperature) to stretch the diaphragm. This resulted in a better distribution of the light. Before placing and fitting the transparent sterile bag, optimal haemostasis was obtained and the thoracic cavity was cleaned thoroughly to prevent light absorption by blood. The light source was introduced via a sterile tube (stomach catheter 18G) in the centre of the filled bag. The surgical wound was approximated during the illumination to maximize back scattering of light due to reflections in the cavity. This procedure was performed to obtain a uniform light distribution. Before and after illumination, the presence of blood pockets was easily verified by visual inspection of the saline in the bag.

The distribution and total dose of the light delivered was monitored with isotropic light detectors (probes) with an accuracy of ± 15% (Van Staveren et al, 1995), manufactured in the Clinical Physics Department of the Daniel den Hoed Cancer Centre. The probes (diameter 1 mm) were connected to photodiodes (Photop UDT-455, Graseby Electronics, Orlando FL, USA), the output of which was A/D converted and displayed and stored on a PC. This system allowed us to do real-time fluence rate and integrated fluence measurements. The isotropic probes measure the total light fluence delivered to the tissue, including both direct incident and scattered light from the tissue bulk. The latter is not measured when flat photodiodes are used, as in some previous studies of PDT in malignant mesothelioma (Pass et al, 1994).

The probes were calibrated in air in an integrating sphere with a well defined diffuse light field (Van Staveren et al, 1995). The integrating sphere and the photodiodes are incorporated in one device that can be connected to the printer port of any regular PC. The probes were then placed in a sterile polyethylene lockable extension tube (i.d. 1–2 mm, Vygon, Ecouen, France) and filled with saline to match the refractive index of the surrounding medium (saline and tissue), which resulted in correct calibration of the isotropic probes. For further details on these calibrations see Marijnissen and Star (1996). Four probes were used to monitor the treatment. The probes (in the tubes) were sutured at strategic spots in the thoracic cavity before the sterile bag with saline was placed in position. One probe was always situated in the sinus, and one in the top of the thoracic cavity. The third and the fourth probes were positioned to cover representative areas of the cavity, including critical structures such as the oesophagus, the pericardium or the lung surface if only a pleurectomy was performed.

At the start of the treatment, the spherical diffuser was placed in the centre of the cavity and the position of the diffuser was manually adjusted until the fluence rate readings on all detector probes

© Cancer Research Campaign 1997

**Figure 1** Chest radiograph (posteroanterior view) of this patient before operation. The arrows indicate the contours of the mesothelioma on the right side were approximately equal. If the detectors showed an imbalance in fluence rate during the treatment, the diffuser was manually repositioned to regain the desired distribution. If a region (e.g. the sinuses) received insufficient light, this region was given top up illumination using a micro-lens fibre after removal of the saline filled bag. The four probes were recalibrated (software-wise) for the absence of the saline (which causes a refractive index mismatch), and left in place to measure the scattered light at other than the directly illuminated regions. The illumination was continued until the required total dose of 10 J cm$^{-2}$ was reached, averaged over all measured sites. Sometimes an imbalance between the total light dose on the four spots was allowed, for instance when the probe was on a spot with a visible tumour mass.

Twenty-four hours after treatment a thoracoscopy was performed (four patients) through the thoracoport with a 0° rigid optic held in a forceps device (Wolf, Germany). Biopsies were taken from apparently normal tissue and from the marked indicator lesions. The tissue was collected in formaldehyde and processed for histology. The thoracoport was replaced by a transparent tube and additional illumination (20 J cm$^{-1}$ at 400 mW cm$^{-1}$ using a 2-cm cylindrical diffuser) was given to the tract in order to kill any remaining tumour cells.

### RESULTS

Five patients were suitable for the combined surgical/PDT treatment. The mediastinoscopy was normal in these patients, there were no contraindications for a pleuropneumonectomy and written informed consent was obtained. Four of the patients had diffuse mesothelioma (confirmed by an expert panel) for which a pleuropneumonectomy was indicated. The chest radiograph of the first patient is shown in Figure 1. One patient with a localized lesion was treated with excision of the pleural tumour and was illuminated with the lung in situ. This patient was found to have a carcinoid tumour instead of a malignant mesothelioma when the excised tumour was analysed (Table 1). A sixth patient referred for the adjuvant PDT therapy proved to be irresectable at operation. The operation was therefore terminated and no additional PDT was given.

The total PDT treatment time was (maximally) 1 h, including placement of the probes and the bag. The integrated cumulative dose (J cm$^{-2}$) is shown as a function of time for all patients in Figure 2A–E and the delivered dose to all sites of measurement is given in Table 2.

In patient 1 the fluence rates on all but one probe were balanced (Figure 2A). The probe in the sinus showed a low fluence rate, indicating that the diaphragm was not properly stretched. The total light dose to the sinus was less than 1 J cm$^{-2}$. Two additional illuminations using a micro-lens were therefore given. The bag was removed and the diaphragm stretched manually, leaving the other probes in situ. The probe near the oesophagus registered an additional light dose of 2.5 J cm$^{-2}$ as a result of the scattered light from the micro-lens illuminations, the other probes showed an additional dose of 1.5 J cm$^{-2}$. The probe located in the sinus was used to measure the additional dose given by the micro-lens. The total dose on the diaphragm, outside the areas that were additionally illuminated, is estimated to be about 1 J cm$^{-2}$ higher than the initial dose of 8.6 J cm$^{-2}$. The average dose for the entire cavity was 10.1 J cm$^{-2}$.

In patient 2 the (deflated) lung was still present in the thoracic cavity. The dose on the lung surface was monitored to avoid excessive normal tissue damage (Figure 2B). The fluence rate on the probes was balanced, except for the probe on the tumour located on the lateral thoracic wall. The tumour surface was additionally

**Table 1** Patient and tumour characteristics

| Patient no. | Age (years) | Sex | Diagnosis | TNM after resection | Side | Comments |
|---|---|---|---|---|---|---|
| 1 | 37 | M | Epithelioid | T3N1M0 | Right | Extensive tumour mass on diaphragm and mediastinum, pretreatment with one course of mitomycin C, vinblastine and cisplatin |
| 2 | 40 | M | Carcinoid | TxN0M0 | Left | Localized tumour in the parietal pleura (9 cm diameter) that was first diagnosed as a MM at thoracoscopy |
| 3 | 59 | M | Epithelioid | T2N1M0 | Right | Large tumour mass on parietal pleura, diaphragmatic sinuses and in the diaphragm |
| 4 | 41 | F | Epithelioid | T1bN0M0 | Left | Tumour located especially at the diaphragmatic sinuses |
| 5 | 54 | M | Epithelioid | T3N0M0 | Right | Tumour diffusely located in the thorax with location in the top that was difficult to remove surgically. |

T1b, tumour extending on both visceral and parietal pleura; T2, tumour with in growth of the lung or diaphragm; T3, tumour encompassing the pericardium; N0, no lymph nodes; N1, positive lymph nodes within the visceral pleura; M0, no distant metastases.

© Cancer Research Campaign 1997

*© Cancer Research Campaign 1997*

**Figure 3** Fluence rate (mW cm$^{-2}$) recorded in time (min) during the illumination of the thoracic cavity of patient 4. Small changes in fluence rate occur during illumination because of respiration and the manual repositioning of the light probe

**Table 2** Total cumulative light doses delivered to various sites in the thoracic cavity.

| | Light dose (J cm$^{-2}$) | | | | |
|---|---|---|---|---|---|
| **Probe position** | **Patient 1** | **Patient 2** | **Patient 3** | **Patient 4** | **Patient 5** |
| Top of thoracic cavity | 9.8 | 9.9 | 8.5 | 8.5 | 8.5 |
| Lung surface | – | 10.5 | – | – | – |
| Retrosternal | 10.2 | – | – | – | – |
| Oesophagus/mediastinum | 11.7 | – | 7.8 | – | – |
| Tumour | – | 12.8 | – | – | – |
| Pericardium | – | – | – | 8.5 | 10.4 |
| Thorax surgical entrance | – | – | 10.5 | 9.1 | 10.2 |
| Diaphragmatic sinus | < 1.0 | 9.0 | 14.4 | 9.4 | 8.5 |
| Extra sinus illumination, anterior and posterior | 8.6 | – | – | – | – |
| Average | 10.1 | 10.0 | 10.3 | 8.9 | 9.4 |

illuminated with a micro-lens. In this situation the scattered light contributed little extra dose to the other sites of measurement. The diaphragm was well stretched and the light dose in the sinus was sufficient. The tumour received 12.8 J cm$^{-2}$, the average for the cavity was 10.6 J cm$^{-2}$.

In patient 3 well-balanced fluence rates were established on all probes (Figure 2C), with, intentionally, a higher fluence rate on the sinus, which received 14.4 J cm$^{-2}$ to the remainder of tumour mass on the diaphragm. The average dose delivered was 10.3 J cm$^{-2}$.

In patients 4 and 5 the fluence rates on all probes were well balanced (Figure 2D and E). Average doses for the cavity were 8.9 and 9.4 J cm$^{-2}$ respectively. Changes in fluence rate during the illumination period for patient 4 is illustrated in Figure 3. Apart from the fluctuations caused by the manual repositioning of the spherical diffuser, the fluence rate on all probes was kept stable at 3–4 mW cm$^{-2}$.

### Post treatment complications and follow-up

There was no 30 day mortality and the duration of the hospital stay was less than 3 weeks for patients 1, 2, 3 and 5 (Table 3).

In patient 1, skin photosensitivity occurred in the intensive care unit because of monitoring with a pulse-oximeter on the index finger. A small grade 2 sunburn resulted from the measurement (which lasted 4 h). (Other patients were monitored intermittently for several seconds on different fingers.) Eleven months after the operation patient 1 was in good clinical condition and had resumed working full time. A CT scan showed no evidence of tumour recurrence in the thoracic cavity. Patient 2 had recovered fully from the operation and resumed working part-time at 10 months after operation. Physical and radiological examination did not reveal any recurrences and the lung function parameters (spirometry and diffusion) were unchanged. The third patient suffered from consti-

© Cancer Research Campaign 1997

**Table 3** Post-surgical complications and follow-up

| Patient no. | Hospital stay (days) | Complication (post-operative) | Follow-up (months) | Current tumour status and long-term side-effects |
|---|---|---|---|---|
| 1 | 18 | Grade 2 sunburn on index finger | 11, alive | No evidence of tumour recurrence |
| 2 | 16 | No complications | 10, alive | No evidence of tumour recurrence, fatigue since operation |
| 3 | 18 | Constipation due to opiates | 7, dead | Diffuse vascular metastasis originating from the thoracoport leading to abdominal metastasis and death |
| 4 | 41 | Rupture of diaphragm requiring rethoracotomy, increased effusion in operated cavity treated with multiple thoracenteses | 10, alive | No evidence of tumour recurrence, limited exercise tolerability, fatigue |
| 5 | 20 | Haematopericardium requiring drainage grade 2–3 skin burn on a 5 cm area in the surgical scar | 9, alive | Lower back pain with normal MRI, no evidence of tumour recurrence, increased susceptibility for infections |

**Table 4** Tumour and normal tissue response to PDT after 24 h

| Patient | Tumour sample | Normal tissue |
|---|---|---|
| 1 | Partly necrotic, partly viable tumour cells, inflammatory cells and oedema | Fat tissue, muscle cells, partly necrotic, oedema |
| 2 | No data | No data |
| 3 | No tumour cells identifiable, inflammatory cells | Inflammatory cells and muscle cells |
| 4 | No tumour identifiable inflammatory cells, oedema | Inflammatory cells and oedema in striated muscle cells |
| 4 at 48 h | No tumour cells identifiable | No necrosis of diaphragm biopsies or of chest wall biopsies |
| 5 | Necrosis of tumour cells, inflammation | Fat tissue and muscle cells with some necrosis |

pation related to the post-operative pain medication. Reduction of the opiates and oral and rectal laxatives resolved this complication. Sixteen weeks post-operatively, diffuse tumour spread was diagnosed in the superficial skin vessels originating from the thoracoscopic scar. He died of local and abdominal tumour spread at 7 months after treatment. No post mortem was performed.

In patient 4, the diaphragm was elevated one day after surgery and thoracoscopic examination was difficult. On day 2 signs of a diaphragmatic rupture had become apparent and a rethoracotomy had to be performed to replace the stomach in the abdominal cavity and to close the diaphragmatic rupture. The margins of the rupture were biopsied and a Marlex covering was used to strengthen the diaphragm. Increased fluid production in the thoracic cavity resulted in shifting of the mediastinum to the right, thus hampering respiration. Repeated punctures (thoracentheses) were necessary to remove the superfluous pleural fluid. Ten days after treatment the patient had increased dysponea and a pulsus paradoxus was noted. On radiological examination (chest radiograph and ultrasound) a pericardial effusion was noted and 500 $cm^3$ of haemorrhagic fluid was removed. Cytological examination revealed no tumour cells and the post-operatively initiated anticoagulant therapy was withdrawn. Further recovery was uneventful and on day 40 after admission she was discharged. Ten months after treatment she was well with no signs of tumour recurrences on CT scan examination. Patient 5 experienced post-operative pain that could be controlled with analgetics. Nine months after treatment he was still tired but had no other complaints. The radiological examination did not show any signs of recurrences.

None of the patients experienced any generalized sunburn effects after discharge.

### Histological examination

In the 4 patients who underwent pleuropneumonectomy, a thoracoscopy was performed one day after PDT and tissue was taken from

**Figure 4** **(A)** Pleural mesothelioma, epithelial type (H&E, 200×, pleuropneumonectomy specimen) of patient 1. **(B)** Necrotic mesothelioma, 24 h after combined treatment (H&E, 200×, biopsy of target lesion) of patient 1

*© Cancer Research Campaign 1997*

apparent normal and indicator lesions. In Table 4 details of the histological specimens are given. In all specimens obtained, an infiltration with polymorphonuclear cells and oedema was observed. This was more marked in the tumour tissue than in the samples obtained from the chest wall where the tumour had been resected completely. Figure 4A shows an example of vital mesothelioma in the resection specimen of patient number 1. In the biopsies of the target lesion (Figure 4B), sampled 24 h after the combined treatment, necrotic residual mesothelioma with an increased number of polymorphonuclear cells was found. The patient who was reoperated for the diaphragmatic rupture also had tissue sampling 48 h after the PDT. Biopsies taken from the diaphragm at the site of rupture showed a thin muscle layer but no evidence of (PDT induced) necrosis was found.

### DISCUSSION

From the time of diagnosis of MM, the mean survival is generally 9–14 months despite aggressive treatment. It is well recognized that surgery alone is insufficient for the majority of malignant pleural mesothelioma cases and (adequate) adjuvant therapies are limited by their toxicity and damage to normal tissue or the inability to cover all sites of the resection areas. Various chemotherapeutic regimens containing mitomycin-C and doxorubicin have been investigated but their effectiveness has not been demonstrated clearly. Although some long term survivors have been reported, the overall effect has been disappointing (Krarup-Hansen and Hansen, 1991). The addition of radiotherapy to the operated hemithorax has also failed to demonstrate an increase in survival (Hilaris et al, 1984; DaValle et al, 1986). One of the major problems is the planning of the radiation field to administer a tumoricidal dose without excessive normal tissue toxicity.

The primary objective of this study was to develop a suitable illumination and dosimetry procedure for intrathoracic PDT. Photodynamic therapy offers some advantages as a local adjuvant treatment as it has a restricted normal tissue toxicity that is related to the depth of penetration of the light used and the concentration of photosensitizer. For optimal efficacy, the photosensitizer should preferentially concentrate in tumour tissue, and/or its vasculature, and its singlet oxygen production should be high on illumination. A second important aspect is that optimal light delivery and dosimetry, especially when larger areas are treated.

Promising results on the use of PDT in MM have been reported in the literature. In the largest study (Pass et al, 1994) 42 patients were treated with haematoporphyrin derivative and illumination in a phase I study, but no increased survival (mean 12.4 months) was observed. The illumination, 2 days after sensitization, was performed with two argon dye lasers. The fluence rate for each laser at 630 nm was 5 W maximally and the average additional time to perform the PDT was 89 min (including placement of fibres). The actual laser time was 68 min to achieve a total light dose of 25 J $cm^{-2}$. As light dosimetry was performed with flat photodiodes, which do not measure all scattered light, the total light delivery to the tissue was underestimated. Ris et al (1996) performed a pilot study in eight patients using 0.3 mg $kg^{-1}$ m-THPC and 10 J $cm^{-2}$ after an interval of 48 h. Seven patients had good local control of their thoracic disease but developed distant metastasis after 4–18 months. One patient died of pulmonary embolism 8 days after resection. Post-mortem examination showed extensive necrosis in the remaining tumour but no damage to normal structures such as the heart and the oesophagus.

In all previous studies with PDT its potential has been well recognized, but the lack of understanding of dosimetry and the inability to administer adequate light doses in a short period of time has prevented a more general use of PDT. The development of second generation photosensitizers, with a higher singlet oxygen yield, have made PDT treatment more attractive, especially for larger areas. The higher activity of the photosensitizers and the new types of high-power diode lasers now available enables the treatment period to be considerably shortened. We have been able to apply this adjuvant photodynamic treatment in 40–60 min. The shorter half life or reduced retention in the skin of such photosensitizers also greatly reduces the skin photosensitivity for the patients.

The cumulative light dose (fluence) to the tissues of the thoracic cavity can be measured by isotropic probes in real-time. This is independent of the optical properties of the tissues in the cavity, which may vary considerably between patients. These differences are enhanced by multiple reflections in a closed cavity with walls of scattering tissue (Van Staveren et al, 1994). The prescribed light dose, based on the use of isotropic probes for in situ dosimetry, will be lower than the same dose specified with the use of flat detectors, because the flat detectors do not detect all the light in the tissue. It is therefore impossible to compare results from different studies on the basis of incident light dose alone. To overcome this problem, generally accepted methods of light delivery and measurement should be developed for specific clinical settings. The total light dose received by the target tissue should be described by in situ dosimetry measurements. With the simple illumination technique used in this study, one spherical diffuser occasionally supplemented with a micro-lens illumination, it was possible to achieve a controlled distribution of the light delivered to the thoracic cavity. The on-line measurement allows for repositioning of the diffuser during the treatment, thereby correcting for asymmetry in the fluence rate distributions and preventing under- or over-illumination of some areas. Great care must be taken when illuminating the cavity sequentially with adjoining light fields, as has been done in several studies (e.g. Ris et al, 1996), as the scattered light contributes a considerable amount to the dose received on locations that are not directly illuminated. This could lead to overdosing of some areas, unless this is continuously monitored during illumination.

In this study we have shown that the combination of PDT and surgical resection for pleural tumours is feasible and that in situ light dosimetry greatly improves the reproducibility and controllability of this combined treatment. The operation time increased by less than 1 h (on an average of 5 h surgery alone) in the current set-up and the duration of the hospital stay was generally no longer than expected for surgery alone. Larger numbers of patients and longer follow-up will be required to determine whether this type of adjuvant treatment offers a benefit in terms of local control and survival. The absence of tumour recurrence and survival, observed in four of five patients so far, is promising. In the patient who died with recurrence after 7 months, tumour cells may have been spread from the thoracoscopic entrance. We have therefore abandoned the practice of leaving an indwelling thoracoport for 24 h biopsy sampling.

Tissue specimens, taken 24 h after treatment, showed extensive damage to the tumour tissue and only minor damage to striated muscle of the chest wall after a light dose of only 10 J $cm^{-2}$ . Clearly the light dose and/or the drug dose could be escalated or the time interval between drug administration and illumination could be reduced in an attempt to achieve better results.

© Cancer Research Campaign 1997

The improvements in methods of light administration and dosimetry greatly improve the possibility of safely applying PDT on larger surfaces. Using this information as a starting point, we are now planning further dose escalation and phase II studies.

### ACKNOWLEDGEMENTS

We thank Dr Willem Star for his comments and advice and Hugo Oppelaar for his technical support and gratefully acknowledge the support of Scotia Pharmaceuticals, Guildford, UK. The study was partly supported by the Dutch Cancer Society (project NKI 97-1446).

### REFERENCES

Butchart EG and Gibbs AR (1990) Pleural mesothelioma. *Curr Opin Oncol* **2**: 352–358

Chahinian AP, Pajak TF, Holland JF, Norton L, Ambinder RM and Mandel EM (1982) Diffuse malignant mesothelioma: prospective evaluation of 69 patients. *Ann Int Med* **96**: 746–755

Chahinian AP, Antman K, Goustou M, Corson JM, Suzuki Y, Modeas C, Herndon II JE, Aisner J, Ellison RR, Leone L, Vogelzang NJ and Green MR (1993) Randomised phase II trial of cisplatin with mitomycin or doxorubicin for malignant mesothelioma by the Cancer and Leukemia Group B. *J Clin Oncol* **11**: 1559–1565

DaValle MJ, Faber LP, Kittle CF and Jensik RJ (1986) Extrapleural, pneumonectomy for diffuse malignant mesothelioma. *Ann Thorac Surg* **42**: 612–618

Hilaris BS, Nori D, Kwong E, Kutcher GJ and Martini N (1984) Pleurectomy and intraoperative brachytherapy and postoperative radiation in the management of malignant pleural mesothelioma. *Int J Radiat Oncol Biol Phys* **8**: 19–25

IMIG (1995) A new staging system for malignant mesothelioma. *Chest* **108**: 1122–1126

Krarup-Hansen A and Hansen HH (1991) Chemotherapy in malignant mesothelioma : A review. *Cancer Chemother Pharmacol* **28**: 391–394

Lofgren L, Larsson M, Thaning L and Hallgren S (1991) Transthoracic endoscopic photodynamic therapy for malignant mesothelioma. *Lancet* **337**: 359

Marijnissen JPA and Star WM (1996) Calibration of isotropic light dosimetry detectors based on scattering bulbs in clear media. *Phys Med Biol* **41**: 1191–1208

Pass HI, Delaney T, Tochner Z, Smith PE, Temeck BK, Pogrebniak HW, Kranda KC, Russo A, Friauf WS, Cole JW, Mitchell JB and Thomas G (1994) Intrapleural photodynamic therapy: results of a phase I trial. *Ann Surg Oncol* **1**: 28–37

Ris HB, Altermatt HJ, Inderbitzi R, Hess R, Nachbur B, Stewart JCM, Wang Q, Lim CK, Bonnet R, Berenbaum MC and Althaus U (1991) Photodynamic therapy with Chlorins for diffuse malignant mesothelioma: initial clinical results. *Br J Cancer* **64**: 1116–1120

Ris HB, Altermatt HJ, Nachbur B, Stewart CM, Wang Q, Lim CK, Bonnet R and Althaus U (1996) Intraoperative photodynamic therapy with mTHPC for chest malignancies. *Las Surg Med* **18**: 39–45

Ruffie P (1993) Mesothelioma chemotherapy. *Eur Respir Rev* **3**(11): 199–203

Rusch VW, Saltz L, Venkatraman E, Ginsberg R, McCormack P, Burt M, Markman M and Kelsen D (1994) A phase II trial of pleurectomy/decortication followed by intrapleural and systemic chemotherapy for malignant mesothelioma. *J Clin Oncol* **12**: 156–1163

Sørensen PG, Bach F, Bork E and Hansen HH (1985) Randomised trial of doxorubicin versus cyclophosphamide in diffuse malignant mesothelioma. *Cancer Treat Rep* **69**: 1431– 1432

Sugarbaker DJ, Heher EC, Lee TH, Couper G, Mentzer S, Corson JM, Collins JJ, Shemin R, Pugatch R, Weissman L and Antman KH (1991) Extrapleural pneumonectomy, chemotherapy, and radiotherapy in the treatment of diffuse malignant pleural mesothelioma. *J Thorac Cardiovasc Surg* **102**: 10–156

Takita H, Mang TS, Loewen GM, Antkowiak JG, Raghaven D, Grajek JR and Dougherty TJ (1994) Operation and intracavitary photodynamic therapy for malignant mesothelioma: A phase II study. *Ann Thorac Surg* **58**: 995–998

Van Staveren HJ, Beek JF, Ramaekers JWH, Keijzer M and Star WM (1994) Integrating sphere effect in whole bladder wall photodynamic therapy: I. 532 nm versus 630 nm optical irradiation. *Phys Med Biol* **39**: 947–959

Van Staveren HJ, Marijnissen JPA, Aalders MCG and Star WM (1995) Construction, quality control and calibration of spherical isotropic fibre-optic light diffusers. *Las Med Sci* **10**: 137–147

© Cancer Research Campaign 1997